# Peroxidase and β-1,3-glucanase synergistic functions strengthen plant cell wall and protect wheat against *Diuraphis noxia* infestation

**DOI:** 10.1007/s00425-025-04759-1

**Published:** 2025-07-04

**Authors:** Siphephelo N. N. Zondo, Mpho S. Mafa

**Affiliations:** Carbohydrates and Enzymology Laboratory (CHEM-LAB), Department of Plant Sciences, University of the Free State, P. O. Box 339, Bloemfontein, 9300 South Africa

**Keywords:** Callose deposition, Lignin cross-linking, Wheat aphids, Papillae, Phloem-feeders, β-1,3-glucanases

## Abstract

**Main conclusion:**

Peroxidase and β-1,3-glucanase functions improved cell wall lignification and reduced callose during plant–pest interactions. Lignification effectively strengthens the cell wall, whilst callose deposition weakens and makes the cell wall porous.

**Abstract:**

In the past two decades, studies have demonstrated that wheat infested with Russian wheat aphid (RWA) changes its biochemical and physiological metabolic functions. These changes include increased β-1,3-glucanases and peroxidase (POD) activity in the resistant cultivars. The POD activity is associated with reactive oxygen species production or quenching. However, the β-1,3-glucanase physiological function is not fully understood. Here, we reviewed the cell wall-related roles of POD and β-1,3-glucanase and their implication on plant biochemical and physiological processes during biotic stress, particularly RWA infestation. We demonstrate that β-1,3-glucanases cell wall isozymes regulate callose accumulation in the resistant wheat cultivar, improving the transport of signalling molecules within RWA-infested plants. In other plant systems, the β-1,3-glucanases’ activity is linked with the formation of the non-penetration papillae (NPP), whilst the POD is associated with the lignification of the NPP. These cell wall modifications deter RWA feeding and improve plant health. In addition, there is increasing evidence that β-1,3-glucan oligosaccharides trigger pattern immune responses during the plant–pest/pathogen interaction, indicating that upregulation of β-1,3-glucanases activity in resistant cultivars can induce this type of defence response during RWA–wheat interaction. We conclude that POD and β-1,3-glucanases’ activities are central to cell wall reinforcements and induction of immune responses in the resistant wheat infested with RWA. Therefore, this work highlights the synergistic effects of POD and β-1,3-glucanases in regulating cell wall modification that strengthens the cell wall, making it impenetrable to pest (including RWA) attacks.

**Supplementary Information:**

The online version contains supplementary material available at 10.1007/s00425-025-04759-1.

## Introduction

### Russian wheat aphid biotypes and their significance in agriculture

Russian wheat aphid (RWA), *Diuraphis noxia* Kurdjumov (Homoptera: Aphididae) is a major pest affecting small grains and grasses globally (Mirik et al. 2007; Saheed et al. 2007a). It negatively impacts wheat (*Triticum aestivum* L.) and barley (*Hordeum vulgare* L.) grain yield and quality (Belay and Araya 2015). The yield loss and poor grain quality lead to economic loss and food insecurity. The RWA uses volunteer wheat and non-cultivated grass species as hosts to survive the dry period between harvest and sowing (Girma et al. 1993; Weiland et al. 2008; Nematollahi 2016; Burger and Botha 2017). The non-cultivated grass species that host RWA between harvest and sowing of small grains may function as a reservoir for new aphid biotypes, which are more virulent to the previously resistant wheat or barley cultivar (Weiland et al. 2008; Nematollahi 2016).

RWA has developed diverse variations within the populations in various parts of the world (Puterka et al. 1992; Botha 2020). The diversity of the RWA population is driven by high mutation rates that result in increased virulence towards a particular wheat cultivar carrying a resistance gene (Bapela and Tolmay 2022). Mutations allow the RWA population to overcome previously established resistance within the wheat cultivar, developing a new biotype (Burger and Botha 2017; Jankielsohn 2019). New biotypes develop due to the deployment of resistant cultivars, which in turn exert selection pressure on the existing biotypes, leading to the development of more virulent biotypes (Botha et al. 2005; Weiland et al. 2008; Jankielsohn 2019; Bapela and Tolmay 2022). Since the discovery of the first RWA biotype in South Africa in 1978, reported by du Toit and van Niekerk (1985), it has undergone several selection pressure events, leading to five distinct RWA biotypes in South Africa (Burger and Botha 2017; Jankielsohn 2019; Botha 2020). The five South African biotypes are designated RWASA1–RWASA5 and mostly were first reported in the Eastern Free State (Tolmay 2006; Tolmay et al. 2007; Jankielsohn 2011, 2018; Botha 2020). It is important not to confuse SA biotypes with the eight biotypes reported in the United States of America (Puterka et al. 2014; Xu et al. 2020; Botha 2020), because the SA biotypes are virulent to wheat cultivars that are resistant to USA biotypes (personal communication with Vicki Tolmay in 2025). 

### Impact of RWA feeding on wheat plant phenotype and vascular tissue

RWA uses a piercing-sucking feeding mode, which generally damages cells by piercing (Franzen et al. 2008). During feeding, RWA injects effectors into the apoplastic regions of the wheat plant, resulting in the development of the symptoms associated with RWA infestation in the wheat plant (Mirik et al. 2007; Jimoh et al. 2011; Mohase and Taiwe 2015). The symptoms of RWA infestation include leaf chlorosis, appearing as white or reddish-purple longitudinal streaks on the leaves and sometimes on the stem. In heavily infested wheat plants, the reduced chlorophyll concentration leads to stunted growth, decreased leaf area and dry weight, and the leaves’ roll (Belefant-Miller et al. 1994; Mirik et al. 2007; Cooper et al. 2010; Mohase and Taiwe 2015; Jimoh et al. 2018). Saheed et al. (2007b) argued that the development of white and yellow streaks was due to the injection of aphid saliva into the xylem. The action of blowing out the stylet ducts seals the xylem and disrupts the phloem, leading to the induction of leaf rolling and streaking due to the isolation of these conducting tissues (Saheed et al. 2007b). These become more evident in susceptible cultivars than resistant ones. Wheat plant responses to aphid infestation were influenced by factors, such as plant growth stages, time and duration of the feeding, nutritional status of the host plants, aphid abundance, and environmental factors (Mirik et al. 2007; Achilonu et al. 2014). 

Russian wheat aphids ingest phloem sap through stylets that penetrate intercellular plant tissues and tap the phloem sieve tube (Botha and Matsiliza 2004; Mohase and Taiwe 2015; Walker 2022). They prefer to pierce the thin-walled sieve tubes (Saheed et al. 2010; Walker 2022), followed by injection of watery saliva, which conditions the sieve element to inhibit phloem-sealing defensive mechanisms, exposing sap for ingestion (Powell et al. 2006; Cooper et al. 2010). Therefore, RWA feeding interferes with the phloem transport capacity in the infested plants (Saheed et al. 2010). Successful feeding occurs in susceptible cultivars; however, resistant cultivars respond to RWA infestation by inducing resistance genes, defence-related proteins, and enzymes responsible for the detoxification of reactive oxygen species, hypersensitive response, and reinforcements of the cell wall (Van der Westhuizen et al. 2002; Cooper et al. 2010; Mafa et al. 2022). Peroxidases were involved in wheat defensive responses against RWA infestation by regulating the oxidative status of the plant cell or cell wall region, as well as strengthening the cell wall through lignin cross-linking and cell wall compounds cross-linking (Van der Westhuizen et al. 1998b; Mohase and Taiwe 2015; Mafa et al. 2022; Zondo et al. 2024a, b).

The ongoing arms race between wheat and RWA biotypes suggests a need to study other defence-related mechanisms that plants use to reduce the impact of RWA infestation. The molecular and gene-related approach to tackle this challenge is addressed in another literature review (Botha et al. 2005; Botha 2020). Here, the physiological and biochemical approaches are used to understand the synergistic effects of the β−1,3-glucanases and peroxidases on the plant cell wall. The synergistic effect allows differentiation of their roles between RWA-infested susceptible and resistant wheat cultivars. For instance, several studies have shown the upregulation of the β−1,3-glucanases and peroxidases in the resistant cultivars infested by RWA (Van der Westhuizen et al. 1998a; Mohase and Van der Westhuizen 2002a, b; Mafa et al. 2022; Zondo et al. 2024a). The induction of these enzymes leads to cell wall modification, amongst other processes, during wheat–RWA interactions.

This literature review attempts to demonstrate that the combined/synergistic actions of peroxidase and β−1,3-glucanase fortify the cell wall of resistant wheat cultivars during RWA infestations. Therefore, we first review biochemical characterisations of peroxidase and β−1,3-glucanase, followed by a clear demonstration that β−1,3-glucanase expression and activity levels in the resistant and susceptible wheat cultivars result in different callose accumulation and regulation during RWA infestation. This information will shed light on the physiological and biochemical approaches that can strengthen environmentally friendly methods to protect wheat against RWA infestation.

### β−1,3-Glucanase and peroxidase accumulation in RWA-infested wheat

Upon RWA infestation, wheat plants activate complex defence responses, including biochemical, physiological, and morphological modifications (Botha et al. 2005; Mohase and Taiwe 2015; Mafa et al. 2022). The direct biochemical defence responses include producing secondary metabolites (such as phenolic compounds or alkaloids) and inhibiting RWA proteolytic and glycoside hydrolase enzymes (Botha et al. 2005; Dornez et al. 2010). In addition, the direct defence responses include induction of the pathogenesis-related (PR) proteins such as β−1,3-glucanase and peroxidase (POD), which facilitate the cell wall reinforcement through callose degradation and lignin cross-linking processes (Van der Westhuizen et al. 1998b; Saheed et al. 2007b).

The β−1,3-glucanase and POD proteins function efficiently under acidic conditions similar to those in the cell wall (Cosgrove 2000, 2022; Mafa et al. 2020; Zondo et al. 2024b). These proteins were secreted into the intercellular space before being trafficked into the plant cell wall region (Stintzi et al. 1993; van Loon 1997; Taiwe 2011). For instance, β−1,3-glucanase and POD activity levels were induced during RWA infestation of resistant wheat cultivars (carrying *Dn* genes), whilst their levels were reduced at early stages of infestation in corresponding susceptible cultivars (Van der Westhuizen et al. 1998b; Saheed et al. 2007b; Mafa et al. 2022; Zondo et al. 2024a).

The apoplastic class III peroxidases are induced to higher levels during hypersensitive response (HR) in resistant plants and are generally associated with various defence-related events. Class III peroxidases are important regulators of extracellular H_2_O_2_ and producers of extremely reactive oxygen species (ROS) (Simonetti et al. 2009). Additionally, class III peroxidases have a broad range of substrate specificity and, therefore, various functions in plant developmental processes, including cell wall stiffening, defence against pests and pathogen infection, tissue damage and H_2_O_2_ removal, and cross-linking the cell wall polymers (Almagro et al. 2009; Kidwai et al. 2020; Su et al. 2020). We propose that the simultaneous induction of β−1,3-glucanases and PODs’ activity levels in the resistant cultivars is directly linked with cell wall reinforcement in the RWA-resistant wheat cultivars. In the next sections of this literature review, we attempt to elaborate on the cell wall-related function of these enzymes.

### The role of β−1,3-glucanase and peroxidase isozymes

Some of the PR proteins play a central role in plant defence responses against pathogens and pests such as aphids (Esquerre-Tugaye et al. 2000). The two most abundant and highly regulated enzymes recognised for defensive roles in wheat plants under stress are β−1,3-glucanases and POD (Van der Westhuizen et al. 2002; Botha et al. 2005; Moravcikova et al. 2016; Mafa et al. 2022). Table S1 summarises a few examples of the plant β−1,3-glucanase and POD isozymes studied from different plant sources. The isozymes were localised in the same or different cell organelles but displayed similar functions and conserved amino acid sequences in the catalytic region and quaternary structural folding (Stintzi et al^1993^; Fernandes et al. 2013; Francoz et al. 2014). In some cases, they displayed different expression levels on western blot or molecular weights on SDS-PAGE. However, they retained a similar function (Hoj et al. 1988; Van der Westhuizen et al. 1998a, 2002; Moravcikova et al. 2016).

Seevers et al. (1971) revealed that some POD isozymes are induced at different plant growth stages and their levels change over time as the plant develops, but isozymes induced by the stem rust infection of resistant wheat cultivar resulted in inhibited fungal development and reduced leaf damage. In addition, POD isozymes'biochemical properties, such as pH and temperature optima, differed between enzymes sourced from different plants, but the functions remained the same. The isozymes’ kinetic properties and substrate specificity may also vary due to their physiological function in a particular cell organelle (Stintzi et al. 1993; Yedidia et al. 2000; Saikia et al. 2005; Ebrahim et al. 2011). The biochemical properties of the β−1,3-glucanase and POD isozymes indicated that in the cereal crops, these enzymes share the locations (cell wall or apoplastic regions), mesophilic temperature, and acidic pH optima. To understand the combined physiological functions of these enzymes in plants, researchers should investigate the synergistic effects of β−1,3-glucanase and POD isozymes on the cell wall region during wheat/barley–RWA infestations.

### Peroxidases cross-link lignin, reinforcing the cell wall during wheat/barley–RWA interaction

Peroxidase activity is usually measured as the oxidation of guaiacol in the presence of H_2_O_2_ (Moloi and Van der Westhuizen 2005; Appu et al. 2021). POD is an important plant enzyme in the biochemical defence mechanisms of plants and is also involved in plant metabolism after tissue damage. Peroxidase is key in defending plants from accumulating harmful ROS (Moloi and Van der Westhuizen 2005; Shigeto and Tsutsumi 2015). The plant’s resistance or susceptibility is associated with the changes in POD activity levels in various plants (Saravanan et al. 2004; Appu et al. 2021). POD enzymes largely act as catalysts in the build-up/cross-linking of the lignin in the plant cell wall, leading to enhanced mechanical strength (Yanti 2015; Mnich et al. 2020).

The zones of lignification and areas of high H_2_O_2_ accumulation in the leaf tissue were used as evidence for peroxidase involvement in cell wall lignification in vivo (Olson and Varner 1993; Ostergaard et al. 2000). One of the POD isozymes was predominantly expressed in lignifying *Arabidopsis* cell suspension, indicating its involvement in cell wall lignification because of its broad substrate specificity in generating and maintaining vascular tissue (Ostergaard et al. 2000; Almagro et al. 2009). Upregulation of POD activity is one of the early biochemical responses in the resistant wheat cultivars infested with RWA, and it was initially thought to be one of the ROS removal systems (Van der Westhuizen et al. 1998a, 1998b; Moloi and Van der Westhuizen 2005). However, there is increasing evidence showing that peroxidase activity in the cell wall region is directly linked to increasing the lignification processes or forming cell wall reinforcing barriers such as papillae in resistant wheat or barley cultivars (Seevers et al. 1971; Yan et al. 2019; Li et al. 2020; Zondo et al. 2024a, b). The cell wall conditions are generally acidic, and most studies have indicated the presence of POD isozymes associated with lignification during RWA–wheat interaction (Ostergaard et al. 2000; Mafa et al. 2022).

The cell wall-bound POD isozyme differs from the apoplastic ones, because it is highly active at pH 5, whilst the apoplastic one is active mainly at pH 6 (Moloi and Van der Westhuizen 2005; Zondo et al. 2024a, b). The role of cell wall-bound POD includes the H_2_O_2_ oxidation and O_2_^−^ production during plant tissue damage, which were identified as defensive responses (Rasmussen et al. 1997; Minibayeva et al. 2009). During excessive stress induced by the excision of roots from wheat seedlings, the cell wall-bound isozymes were released into the apoplast where they prevented ROS accumulation (Minibayeva et al. 2009). Thus, POD functions during wheat/barley–RWA interaction are to balance the oxidative state of the apoplast and the cell wall and to cross-link lignin in the cell wall (Van der Westhuizen et al. 1998a, b; Moloi and van der Westhuizen 2005; Mafa et al. 2022; Zondo et al. 2024b).

### Stress-induced plant β−1,3-glucanase isozymes

Plant β−1,3-glucanases are referred to as the “PR-2 group” of the PR proteins (Table S1), classified into five classes. Classes I and IV are vacuolar alkaline proteins accumulating in mature leaves and roots upon pests/pathogen infection. Classes II and III are acidic extracellular proteins (Moravcikova et al. 2016; Taif et al. 2019; Rajninec et al. 2021). Class IV β−1,3-glucanases are similar to class II but are inducible following tissue damage (Moravcikova et al. 2016). Wheat β−1,3-glucanases studied by Moravcikova et al. (2016) identified six isozymes of which two were active in acidic conditions. Esquerre-Tugaye et al. (2000) identified up to four β−1,3-glucanase isozymes ranging from ~ 30 to ~ 150 kDa in the wheat breeding line SK-196 after tillering, and three isozymes ranging from ~ 30 to ~ 60 kDa, connected to callose deposition and degradation following tissue damage. In addition, β−1,3-glucanase in the vacuole is proposed to be involved in the mediation of the early release of β-glucan elicitors, which interact with/target membrane receptors to initiate defence responses (Esquerre-Tugaye et al. 2000). Muench-Garthoff et al. (1997) reported the accumulation of a 30 kDa β−1,3-glucanase isozyme involved in wheat plant resistance against leaf rust, and another 33 kDa isozyme was reported to accumulate in barley with identical induction for powdery mildew resistance (Jutidamrongphan et al. 1991; Muench-Garthoff et al. 1997).

High levels of β−1,3-glucanase isozyme fraction were purified in the germinating barley, which had an optimum pH of 5.6 (Hoj et al. 1988). This isozyme hydrolysed laminarin substrate to produce oligosaccharides, including laminaritetraose, laminaritriose, laminaribiose, and glucose, (Hoj et al. 1988). In wheat, β−1,3-glucanase isozymes accumulated more in resistant wheat lines infested with RWA than the susceptible ones (Mohase and van der Westhuizen 2002a, b). The concentration levels of these isozymes were lower in the susceptible cultivars compared to resistant ones, and the studies that tracked their activity over an infestation period showed that in susceptible cultivars, β−1,3-glucanase activity levels were reduced within 72 h of infestation (Mafa et al. 2022; Zondo et al. 2024a, b). However, in the resistant cultivar, the activity levels of the β−1,3-glucanase were generally higher than controls from the early infestation periods, and they were higher than in the susceptible cultivars. Furthermore, accumulated physicochemical data show that the isozymes that constitute plant defence responses are highly acidic (pH 3.5–5.5); whilst those observed under the neutral range (pH 6.0 to 7.5) are not induced in the resistant cultivar, but their levels are detected in susceptible cultivars (Taiz and Jones 1970; Van der Westhuizen et al. 1998a; Westhuizen et al. 1998b).

The accumulation of the β−1,3-glucanase in wheat plants under attack by biotic stress agents such as RWA confirms that the enzyme is associated with defence in the resistant plant. Also, suppressing the β−1,3-glucanase enzyme activity in the susceptible plant indicated that this enzyme is significant for the wheat plant survival during the RWA infestation. Yet, the mechanism of action is not fully understood, and what makes attempts to study the mechanism of action challenging is the numerous identified β−1,3-glucanase isozymes during pest–wheat interaction. However, acidic β−1,3-glucanase isozymes are more appealing for further investigations in the biochemistry and pre-breeding fields to fully elucidate the mechanism of action, leading to resistance against biotic stress in wheat. The acidic isozymes are more likely to be localised in the plant cell wall region as shown by Zondo et al. (2024b), in a method developed to extract cell wall-associated β−1,3-glucanase.

###  β−1,3-glucanases produce bioactive oligosaccharides from β-glucan

The β−1,3-glucans are major components of some fungal cell walls and are also present in wheat leaves (and seeds) at lower concentrations compared to starch, cellulose, and xylan (Saulnier et al. 2012; Chowdhury et al. 2014; Wang et al. 2021a). During seed germination, β−1,3-glucan was reported to be hydrolysed by β−1,3-glucanase enzymes to produce oligosaccharides and glucose associated with signalling processes that induced defence response (Hoj et al. 1988; Caruso et al. 1999; Wanke et al. 2020; Perrot et al. 2022). The β−1,3-glucan is also a component of the fungal or bacterial cell wall, and artificially spraying plants with β−1,3-glucan oligosaccharides or microbial inoculum also induced higher levels of β−1,3-glucanase in the treated plants (Anusuya and Sathiyabama 2014; Moravcikova et al. 2016; Perrot et al. 2022). These show that β−1,3-glucanase is directly involved in the defence responses by degrading microbial cell walls or indirectly by producing bioactive compounds.

Walker (2022) reported that RWA secretes saliva containing β−1,3-glucanase into the sieve elements during wheat infestation. The RWA β−1,3-glucanase degrades callose deposited on the layers of the sieve tubes and perforated plates, whilst RWA attack on the plant led to the upregulation of the β−1,3-glucanases in resistant wheat cultivars, a potentially effective strategy for callose regulation preventing phloem clogging. The in vivo studies confirmed that RWA saliva contains the β−1,3-glucanase, which showed activity on the β−1,3-glucan substrates (Mafa et al. 2022). However, it is still not clear whether the RWA saliva contains the β−1,3-glucanases that hydrolyse the β−1,3-glucosidic bonds found in the mixed-linked β−1,3–1,4-glucan or are specific for callose β−1,3-glucosidic bonds. The mixed-linked glucan is part of the hemicellulose component of the cell wall in wheat or other monocot plants. It would be interesting if future research could investigate the β−1,3-glucanases activity and biochemical characterisation and determine its mode of action on the soluble or insoluble mixed-linked β−1,3–1,4-glucan substrates. In addition, similar studies should be conducted during RWA–wheat or RWA–barley interaction to determine if the plants can produce the β−1,3-glucanase enzymes that hydrolyse the soluble or insoluble mixed-linked β−1,3–1,4-glucan substrates.

During RWA infestation, the plant cell wall is weakened in the susceptible wheat cultivar. Cell wall damage in the susceptible wheat is associated with higher activity of cell wall-degrading enzymes (CWDE: including β−1,3-glucanase) that hydrolyse different polysaccharides that constitute plant cell wall, such as callose, cellulose, hemicellulose, and pectin (Zipfel 2009; Pontiggia et al. 2020; Wang et al. 2021a)*.* Mafa et al. (2022) demonstrated that RWASA2 saliva contained CWDEs showing different activities, i.e., cellulase, xylanase, and pectinase activity. The CWDEs produced different fragments patterns on the TLC plate confirming that the RWASA2 is capable of inducing cell wall damage by hydrolysing different polysaccharides of the cell wall. Other studies argued that released oligosaccharide fragments with degrees of polymerisation (DP) between 2 and 10 act as elicitors that induce signal transduction in the resistant plants and further stimulate the plant’s immune response to enhance resistance to pests and pathogens, including RWA (Forslund et al. 2000; Wang et al. 2021a, b). Some of the recorded oligosaccharides with DP of 6 glucose units [e.g., β−1,3-D-(Glc)-hexasaccharide] were considered classified as damage-associated molecular patterns (DAMPs) (Wu et al. 2014; Melida et al. 2018; Pontiggia et al. 2020). During RWA infestation, callose accumulation at the site of plant cell wall damage was degraded by the action of β−1,3-glucanase producing DAMPs. Other studies revealed that DAMPs can activate pattern-triggered immunity (PTI), which is one of the plant defence mechanisms against biotic stress (Wu et al. 2014; Pontiggia et al. 2020; Wang et al. 2021a, b). Even though there is no imperial evidence on the quantification of the DAMPs or induction of PTI during the RWA–wheat interaction, mounting evidence shows a lack of callose degradation in the susceptible cultivar and effective induction of β−1,3-glucanase that degrades callose (potentially producing DAMPs/bioactive molecules) in the resistant cultivars (Forslund et al. 2000; Saheed et al. 2009).

## Callose deposition and papillae formation

### Callose chemical composition and depositions

Callose is the β-(1–3)-linked-d-glucan, which is a carbohydrate polysaccharide present as the component of the cell walls of some cereals and grain crops at different stages of growth and differentiation (Chen and Kim 2009; Pirselova and Matusikova 2012). It is abundant in the monocots and economically important cereal crops, including barley, wheat, and rice. This polysaccharide is found in plasmodesmata canals, root and stem hairs, and vascular tissues (Nakashima et al. 2003; Cierlik 2008; Saheed et al. 2008; Chen and Kim 2009). The callose deposited in wheat cultivars was reported to participate in various structural and physiological functions. For example, (1) in the sieve plates and cell plates, callose is involved in the formation of the plates during late cytoplasmic division (Thiele et al. 2009; Drakakaki 2015); (2) or it is also involved in regulating plasmodesmata permeability by controlling symplastic trafficking; (3) or during pollen tube development, they provide the plant cell with mechanical resistance to tension and prevent sieve element leakage post-tissue damage (Wu et al. 2018; Silva-Sanzana et al. 2020).

Generally, β-glucan dissolves in water and dimethylsulfoxide (DMSO), and the one derived from barley possesses these properties (Tsuchiya et al. 2005; Ramesh and Tharanathan 2003). The callose chemical structure in plants consists of β−1,3-d-glycosidic or mixed-linked β−1,3–1,4-glycosidic bonds connecting glucose molecules, which further causes overlapping of chains in helical form (Nakashima et al. 2003; Cierlik 2008; Rahar et al. 2011). In the linear mixed-linked β−1,3–1,4-d-glucans, β−1,4-linked-glucan units are separated by a single β−1,3-linkages after every three or four glucose residues (Lazaridou and Biliaderis 2007). The callose/β−1,3-glucan is also present in multicellular green algae, fungi, and laminarin, where it is made up of β−1,6-branches (Chen and Kim 2009; Rahar et al. 2011; Wang et al. 2021b). The β-(1,3)-glycosidic bonds make the backbone of the chain and β−1,6-linkages are minor components at a 3:1 ratio (Ramesh and Tharanathan 2008). The β-glucan derived from yeast and mushrooms contains β−1,3-glucan linkages and occasionally β−1,6-branches, whereas the one derived from yeast is a mixed-linked β-(1,3)-(1,6)-glucan (Zavaliev et al. 2011; Rahar et al. 2011).

Callose significantly accumulated in barley leaf tissue infested with RWA over 14 days (Saheed et al. 2009). The authors demonstrated that callose was deposited as early as 24 h after infestation and reached higher levels between 7 and 14 days post-infestation (dpi). After 14 dpi, callose deposition was not increasing in the infested leaf tissue. In contrast, the same study found that the bird cherry-oat aphid (BCA: *Rhopalosiphum padi* L) infestation only induced slightly higher levels of callose in the barley leaf tissue compared to the control. Callose was heavily deposited around the RWA stylets, indicating that RWA saliva contained the effectors that induce the deposition of this polysaccharide in the infested susceptible wheat and barley samples (Botha and Matsiliza 2004; Saheed et al. 2009). In the following sections, we discuss why callose deposition is part of a susceptibility factor during RWA wheat or barley infestation.

### Callose/papillae: physical barrier and implication to defence responses

Callose production in plants is generally associated with cell wall reinforcement, which limits the penetration of pathogens in the cell (Abou-Saleh et al. 2018). Saheed et al. (2007b) demonstrated that RWA infestation induced extensive callose deposits that disrupted plasmodesmata in susceptible wheat (Betta cultivar). In contrast, the resistant cultivar (Betta-*Dn*1) displayed lower levels of callose deposition and reduced plasmodesmata disruption. The authors argued that RWA probing in the susceptible cultivar-damaged sieve tubes and perforated plate cells induced callose deposition to the point of clogging the wounded area, and the development of ectodesmata (Saheed et al. 2007b). In addition, other studies argued that the damage caused by RWA during feeding and probing does not induce callose rather the saliva injected by the stylet in the phloem or cell wall induces callose in susceptible wheat/barley cultivars (Botha and Matsiliza 2004; Saheed et al. 2009). Callose accumulation interferes with the function of the plant’s assimilates transport systems in the infested susceptible wheat or barley cultivars by clogging the xylem and phloem (Botha and Matsiliza 2004; Saheed et al. 2007b, c, 2009). In addition, clogging of plasmodesmata affects the transport of the signalling molecules, perhaps leading to susceptibility in the RWA-infested barley/wheat.

**Fig. 1:**
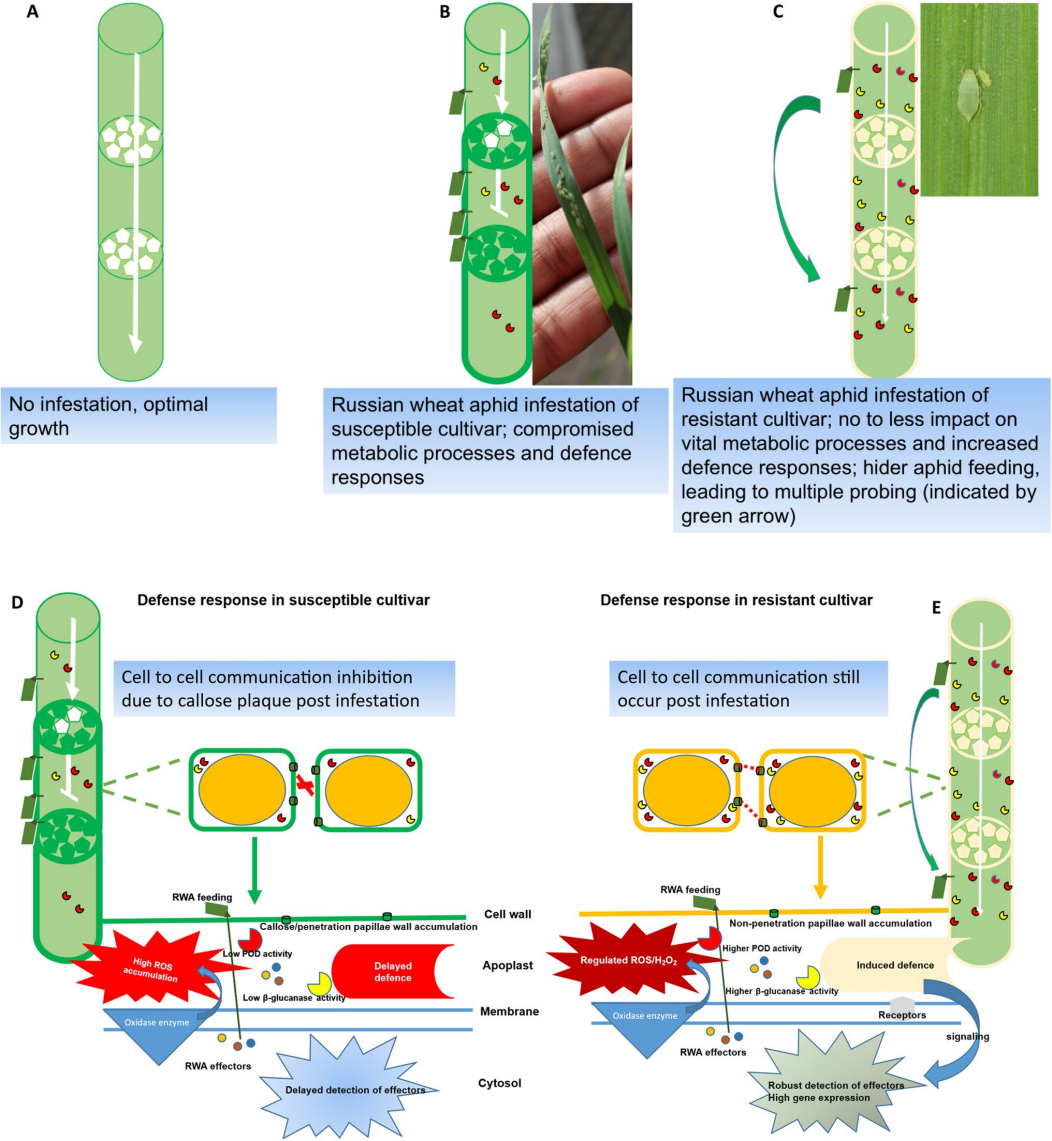
RWA infestation of wheat leads to differential defence responses in susceptible and resistant cultivars. **A** represents a plant that is not infested by RWA, and its growth is optimal. **B**,** D** Represents susceptible wheat infested with RWA, and due to infestation, callose is deposited in the inner walls of the phloem conduits and perforated plates (indicated by green pentagons and bold conduit lines). The callose mass (penetration papillae) accumulation clogs perforated plates (indicated by a white inverted T-shape), increasing the concentration of photoassimilates at the clogged section of the conduits, which leads to higher aphid populations feeding at this point. Due to the susceptible nature of the wheat cultivar, the defence responses are delayed or reduced, e.g., reduced β-glucanase and peroxidase, and cell-to-cell communication is blocked by the callose plaque that clogs plasmodesmata. **C**,** E** represents the resistant wheat–RWA interaction, and due to infestation, the plant induces the callose deposition coupled with induced defence responses. The increased β−1,3-glucanase and peroxidase activities are part of the defence responses, whereby the β-glucanase activity regulates callose deposition, resulting in lesser accumulations compared to the susceptible cultivars. This reduced/regulated callose deposition allows for cell wall reinforcement, leading to the formation of non-penetration papillae at the site of RWA infestation. In addition, increased peroxidase activity cross-links lignin or phenolic compounds to the holocellulose component of the cell wall, reinforcing the cell wall (indicated by the light-yellow conduit with medium bold lines). The white narrow arrow indicates that photoassimilates can flow through the phloem perforated plates, suggesting that a complex cross-linking process between callose, holocellulose, and lignin from the non-penetration papillae, which hinders feeding of the RWA, and allows cell-to-cell communication. Increased peroxidase activity also removes the reactive oxygen species (ROS) in the apoplast and cell wall regions

Previous studies have reported that callose deposition plays a significant role as a universal protection mechanism against phloem-sucking pests, including RWA (Huckelhoven 2014; Walker 2022). This claim was supported by callose’s ability to clog phloem elements, preventing the phloem sap ingestion and assimilates loss after the aphid stylets have disrupted/pierced the sieve elements (Saheed et al. 2008; Silva-Sanzana et al. 2020). However, based on other studies and our experiences, we propose that the callose clogging of the phloem elements and plasmodesmata leads to susceptibility of wheat/barley during infestation. RWA feeding was reported to cause an extensive callose deposition in the phloem channels on susceptible wheat, which impedes the flow of the phloem sap. This allows the development of local sinks for photoassimilates storage (Botha and Matsiliza 2004; Walker 2022). We suspect that the clogging of the phloem increases the concentration of photoassimilates, which are the food source for aphid feeding in susceptible plants. Hence, the RWA feeding on susceptible wheat cultivars multiplies quickly, and plants show symptoms of infestation. Furthermore, callose plugs the plasmodesmata in susceptible cultivars and blocks the movement of essential signalling molecules from one cell to another (Ellinger and Voigt 2014). The implications of these non-regulated, high callose accumulations in the susceptible cultivars is that vital plant communication processes are blocked, leading to a delay or lack of active defence-related biochemical pathways associated with susceptible wheat or barley plants (Fig. 1).

Other studies also support the hypothesis that during the RWA–wheat/barley interaction, high callose accumulation leads to plant susceptibility (Botha et al. 2005; Tilsner et al. 2016; Silva-Sanzana et al. 2020; Mafa et al. 2022). For instance, high levels of callose deposition were reported to clog the plasmodesmata in susceptible cultivars, but low deposition levels in resistant lines allowed the cell-to-cell communication and photoassimilates transport processes (Ellinger and Voigt 2014; Tilsner et al. 2016; Wang et al. 2021b). In addition, more virulent RWA biotype induces more callose deposition in wheat cultivars (e.g., RWASA2 is more virulent than RWASA1), indicating that a significant reduction of callose accumulation was associated with resistance to RWA infestation (De Wet and Botha 2007; Jimoh et al. 2011). We attribute the lower levels of callose deposition in the resistant wheat or barley cultivars infested with RWA to the increased levels of β−1,3-glucanase activity, which gradually increase over the infestation period. Several studies showed that these enzyme activity levels were always higher in the resistant wheat cultivar than in the susceptible one (Van der Westhuizen et al. 1998a; Van der Westhuizen et al. 2002; Moloi and van der Westhuizen 2005). This led us to speculate that infested plants could produce the highly increased β−1,3-glucanase activity to regulate and reduce callose accumulations in the plasmodesmata and vascular tissues (Fig. 1).

### Can papillae formation distinguish resistant cultivars from susceptible ones?

Studies confirm that the susceptible and resistant wheat or barley cultivars can deposit the callose during the RWA infestation. However, they have only focussed on callose accumulation levels. To the best of our knowledge, no studies have demonstrated the composition of the callose, i.e., papillae (β−1,3-glucan mixed with lignin and holocellulose) formation in the resistant cultivars. Papillae is a dome-shaped apposition produced between the epidermal wall and plasma membrane that forms part of the frontline defensive responses reinforcing the plant cell wall near the site of penetration by pests/pathogens (Chowdhury et al. 2014; Silva-Sanzana et al. 2020). Two types of papillae form due to biotic stress: the non-penetration papillae (NPP) and penetration papillae (PP) (Chowdhury et al. 2014; Huckelhoven 2014). The NPP is composed of higher callose, arabinoxylan, and phenolic content (usually linked to lignin), and is induced in the early responses to biotic stress. Phenolic components, such as ferulic and p-coumaric acids in the papillae, allow for covalent cross-linking of phenolic acids and arabinose substitutions of arabinoxylan that account for substantial cross-linking and strengthening (Philippe et al. 2006; Chowdhury et al. 2014). Chowdhury et al. (2014) showed that the NPP composition changes several hours after the initial attack by biotic stress agents, and it mainly contains higher holocellulolytic content (cellulose and arabinoxylan) and lignin content but lower amounts of callose. The callose seems to seal the area damaged by biotic stress agents before growing into a mass that at the centre papillae cross-links with lignin and hemicellulose (arabinoxylan). The outer layer covering the callose consists of holocellulolytic contents.

The NPP were abundant in the resistant barley cultivar infected with *Blumeria graminis* f. sp. *hordei*, whilst the PP was dominant in the susceptible cultivar infected with the same pathogen (Chowdhury et al. 2014; Huckelhoven 2014). It is important to note that the PP lacks the holocellulolytic content, even after a prolonged infection. Overexpression of the *Glucan synthase-Like 5* (*Gsl5*) gene in host plants is essential for penetration resistance as it ensures the production of callose as a key component in NPP at early stages of biotic stress attack (Ellinger et al. 2013; Chowdhury et al. 2014; Ellinger and Voigt 2014; Wang et al. 2021b). Saheed et al. (2009) argued that the role of *GSLs* towards callose synthesis during RWA infestation must be at the protein level because out of 8 *GSLs* genes, only three (Hv.4615, Hv.4615, Hv.17389) that code for the *GSLs* with higher identity to *AtGSL6* were not overexpressed in the susceptible barley cultivar. In addition, the *HvGSL1* gene similar to *AtGsl10* was not expressed in the infested susceptible barley cultivar. The eight genes were not similar to *AtGsl5* (Saheed et al. 2009), which could explain the susceptible nature of this barley cultivar to RWA infestation. Analysis of Saheed et al.’s (2009) findings confirmed that *GSLs* can also be used as a point of reference to distinguish NPP from PP. The absence or lack of *Gsl5* overexpression at the gene or protein levels led to the formation of PP in the RWA-infested wheat plants. The overexpression of these genes identified in the resistant cultivars generally blocks the penetration of pathogen/pest-feeding structures more effectively than in the susceptible hosts. However, NPP should not be viewed as the only source of defence responses against feeding structures that penetrate the cell wall to access nutrients, because there are other alternate defence mechanisms in resistant plants (Zeyen et al. 2002; Chowdhury et al. 2014).

The chemical composition of the NPP and PP leads to a few questions regarding the RWA–wheat interactions. (1) Is the NPP form possibly induced/synthesised in the resistant cultivar upon RWA infestation? (2) Does the upregulation of the peroxidase in the resistant wheat cultivars facilitate the cross-linking of the phenolics with the callose and hemicellulose (arabinoxylan in the case of wheat)? It has been demonstrated that the cell wall gets reinforced in the resistant cultivars during the RWA infestation, particularly the holocellulolytic content and lignin increased in this cultivar (Mafa et al. 2022). Whilst the susceptible cultivar displayed reduced holocellulolytic and lignin contents but very high callose deposition (Saheed et al. 2008, 2009; Mafa et al. 2022). The current data are insufficient to formulate any conclusion on this formation of the NPP in the RWA-infested resistant wheat cultivar. Hence, more studies are needed to investigate the formation of the NPP in the resistant cultivars and compare its chemical composition to that of the susceptible cultivars.

## Conclusion

The literature consulted alludes to the possibility/probability that the β−1,3-glucanases and PODs are involved in essential processes that enable wheat and barley plants to adapt to biotic stresses, including RWA infestation. The β−1,3-glucanases and PODs associated with the defence response against RWA infestation tend to accumulate in the resistant cultivars. Both enzymes enhance cell wall modifications and reinforcement that could deter RWA feeding, whilst their suppression or absence leads to cell wall degradation in susceptible cultivars. The PP is suspected to accumulate at the RWA feeding damaged leaf areas and is expected to be more in susceptible than resistant wheat cultivars. In resistant cultivars, the upregulation of β−1,3-glucanases activity can hydrolyse callose into oligosaccharides, and some of these oligosaccharides are proven to be DAMPs that activate PTI.

NPP is resistant to pest and pathogen penetration and is characterised by callose, cellulose, hemicellulose, and lignin components. It is mostly associated with cell wall modification in plants resistant to biotic stress. This study provided evidence that cell wall modification in a resistant wheat cultivar reduces the intensity of RWA infestation and improves plant health. However, excess unregulated callose deposition results in the formation of PP that weakens the cell wall, leading to extensive feeding in the wheat/barley susceptible plants during the RWA infestation. The lignin cross-linking in the NPP forms due to the overexpression and high activity of POD, resulting in lignification that strengthens the cell wall and sealing leaks caused by the RWA stylet. Therefore, peroxidase activity directly benefits wheat or barley crops by protecting them against RWA infestation.

This literature review shows that the upregulation of POD and β−1,3-glucanase enzymes found in the cell wall and apoplast regions are significant for the production of DAMPs (signalling molecules), lignin cross-linking, regulation of callose deposition in phloem and plasmodesmata, and cell wall reinforcements. The significance of these processes to plant survival explains why the resistant cultivars always induce POD and β−1,3-glucanase at the same time upon RWA infestation. Also, the delayed induction of these enzymes in the RWA susceptible wheat cultivars has been concluded to lead to the development of symptoms and ultimate plant death upon the RWA feeding. This study expands our understanding of the co-expressed protein (POD and β−1,3-glucanase) and their synergistic function that modifies the plant cell wall, showing that the cell wall is not just a rigid physical barrier but is a more dynamic structure that is modified from time to time to protect plants against biotic stress agents.

### Future prospectives

Based on the data presented in this review, it is proposed that peroxidases can reduce aphid feeding on wheat or barley by improving wall stiffening. This process can give plants under attack time to mobilise other defence responses against the aphid attack. Further studies are required to validate some of the claims made in the current literature review about the role of POD on the cell wall during plant–aphid interaction.

## Supplementary Information

Below is the link to the electronic supplementary material.

Supplementary file1 (DOCX 23 KB)

## Data Availability

Data sharing is not applicable to this article as no datasets were generated or analysed during the current study.

## Abbreviations

CWDE: Cell wall-degrading enzymes
DAMPs: Damage-associated molecular patterns
DP: Degrees of polymerisation
*GLS*: *Glucan Synthase-Like* Gene
POD: Hydrogen peroxide (H_2_O_2_) peroxidase
NPP: Non-penetration papillae
PP: Penetration papillae
PR: Pathogenesis-related
ROS: Reactive oxygen species
PTI: Pattern-trigged immunity
RWA: Russian wheat aphid
