# Supplementary material for: Peroxidase and β-1,3-glucanase synergistic functions strengthen plant cell wall and protect wheat against Diuraphis noxia infestation
Source: Planta. 2025 Jul 4;262(2):41. doi: 10.1007/s00425-025-04759-1 (PMC12227470; doi:10.1007/s00425-025-04759-1)
Supplement: Supplementary file 1 — Supplementary file1 (DOCX 23 KB) [file 425_2025_4759_MOESM1_ESM.docx]

Table S1. Classification of pathogenesis-related proteins involved associated with plant defensive responses and their function.

| **PR Group** | **Name of PR Protein** | **Localisation in a cell** | **Functions** | **Molecular weight** | **Reference** |
| --- | --- | --- | --- | --- | --- |
| PR-1 | Cysteine-rich secretory protein, antigen 5 and pathogenesis-related-1 (CAP) proteins | extracellular/apoplastic space | Antifungal activity (Oomycetes) | 15–17kDa | Ferreira et al. 2007; Akbudak et al. 2020 |
| PR-2 | β-1,3-glucanase | Apoplast | Callose degradation | 30–60kDa | Esquerre-Tugaye et al. 2000 |
| PR-3 | Chitinases class I, II, IV, VI, VII | Fungal cell wall | Antifungal activity; Indirect antimicrobial by the release of elicitor-active oligosaccharides | 35–46kDa | Stintzi et al. 1993; Ferreira et al. 2007 |
| PR-4 | Chitin-binding proteins | Fungal cell wall | Antifungal activity | 13–14.5kDa | Ferreira et al., 2007 |
| PR-5 | Thaumatin-like protein | Extracellular & Vacuolar localisation | Antifungal activity | 16–22kDa | Van Loon & Van Strien 1999; Wong & Ng 2011 |
| PR-6 | Proteinase inhibitor | Intracellular | Defense against herbivores and nematodes; Inhibit other enzymes such as α-amylases | >15kDa | Hellinger and Gruber 2019 |
| PR-7 | Endoproteinases | Chloroplast | Microbial cell wall dissolution | 69–70kDa | Vera and Conejero 1988; Taiwe 2011 |
| PR-8 | Chitinase class III | Fungal cell wall | Antifungal activity | 30–35kDa | Ferreira et al. 2007 |
| PR-9 | Peroxidase | Apoplast | Plant cell wall strengthening, limiting pests ingression | 50–70kDa | Stintzi et al. 1993; Passardi et al. 2004 |
| PR-10 | Ribonuclease | Cytoplasm | Plant defense and development; Antiviral activities | 18–19kDa | Yan et al. 2008 |
| PR-11 | Chitinase class V | Fungal cell wall | Antifungal activity | 40kDa | Ferreira et al. 2007 |
| PR-12 | Defensin proteins | Epidermal & Stomatal cells | Antifungal and antibacterial activity | 5kDa | Terras et al. 1992; Van Loon & Van Strien 1999; Parisi et al. 2019 |
| PR-13 | Thionin proteins | Cell walls of cereal plants | Antifungal and antibacterial activity | 5–7kDa | Van Loon & Van Strien 1999; Wong & Ng 2011 |
| PR-14 | Lipid-transfer protein | Extracellular | Antifungal and antibacterial activity | 9kDa | Van Loon & Van Strien 1999; Liu et al. 2015 |
| PR-15 | Oxalate oxidases | Extracellular matrix | Have superoxide dismutase activity | 22–25kDa | Ferreira et al. 2007; Taiwe 2011 |
| PR-16 | Oxalate oxidase-like protein | Extracellular matrix | Have superoxide dismutase activity | 100kDa | Ferreira et al. 2007; Taiwe 2011 |
| PR-17 | NI | Extracellular | Provide enhanced fungal resistance | 23–26kDa | Christensen et al. 2002 |

^a^ NI = Non-identified
